# Linearized Programming of Memristors for Artificial Neuro-Sensor Signal Processing

**DOI:** 10.3390/s16081320

**Published:** 2016-08-19

**Authors:** Changju Yang, Hyongsuk Kim

**Affiliations:** Department of Electronic Engineering, College of Engineering at Chonbuk National University, 567 Baekje-daero, Deokjin-gu, Jeonju-si, Jeollabuk-do 54896, Korea; ychangju@jbnu.ac.kr

**Keywords:** weight programming, complimentary action, anti-serial architecture, linearity weight programming, complimentary action, anti-serial architecture, linearity

## Abstract

A linearized programming method of memristor-based neural weights is proposed. Memristor is known as an ideal element to implement a neural synapse due to its embedded functions of analog memory and analog multiplication. Its resistance variation with a voltage input is generally a nonlinear function of time. Linearization of memristance variation about time is very important for the easiness of memristor programming. In this paper, a method utilizing an anti-serial architecture for linear programming is proposed. The anti-serial architecture is composed of two memristors with opposite polarities. It linearizes the variation of memristance due to complimentary actions of two memristors. For programming a memristor, additional memristor with opposite polarity is employed. The linearization effect of weight programming of an anti-serial architecture is investigated and memristor bridge synapse which is built with two sets of anti-serial memristor architecture is taken as an application example of the proposed method. Simulations are performed with memristors of both linear drift model and nonlinear model.

## 1. Introduction

Memristor is a new circuit element postulated by Leon Chua in 1971 [1] and fabricated recently by the Stanley Williams group [2] from Hewlett-Packard (HP). It exhibits excellent features of both memory [3,4,5,6] and neuromorphic applications [2,7,8,9,10,11]. For memory applications, it is nonvolatile and has an extremely small size of a few nanometers [3,4,5]. For neuromorphic applications, it has features of pulse-based operation and adjustable resistance, which are ideal for tuning the synaptic weights of neuromorphic cells [2,7,8,9,10,12,13].

A convenient way of programming a memristor with a certain value is by applying a rectangular voltage pulse whose magnitude of voltage is constant during pulse period and zero during non-pulse period. However, the variation of its resistance is a nonlinear function of the applied voltage pulse width even it is a linear model. We call the resistance of memristor memristance.

Fortunately, when two memristors with opposite polarities are combined together, the nonlinearity of memristance is reduced dramatically due to the complementary action of two memristors. Kim et al. presented an efficient weighting circuit for synaptic operation by building a bridge structure combining two opposite anti-serial memristor circuits [7].

Waser’s group reported a fabrication result of complementary resistive switch (CRS) consisting of two back-to-back (anti-serial) memristive elements for the construction of large passive crossbar arrays by solving the sneak path problem [14]. Later, the CRS architecture has been further investigated via an analytical approach [15]. T. Liu et al. also reported the Current-Voltage (I-V) characteristics of antiparallel resistive switches (APRS) that strongly depend on the parameters of the individual switches [16].

In this paper, we propose a linearized programming method utilizing an anti-serial architecture. To program a target memristor, the same type of a subsidiary memristor is prepared and connected to the target memristor in series with opposite polarity. Since composite memristance of the anti-serial circuit is a constant value, the current through the circuit is constant. It follows that the memristance variation of the individual memristor is a linear function of pulse width since the memristance variation is a linear function of charge.

The principle of such linear programming is explained theoretically and verified via simulations in this paper. Section 2 describes a reason that memristor is a useful element for building a neural synapse. Section 3 demonstrates nonlinearity in programing of a memristor with rectangular voltage pulses. To resolve the nonlinearity problem of voltage controlled memristor, an anti-serial architecture is proposed in Section 4. Simulation results to support the proposed idea are provided in Section 4. Section 5 is the conclusion.

## 2. Memristor as a Promising Element for the Implementation of Neural Synapses

**Figure 1:**
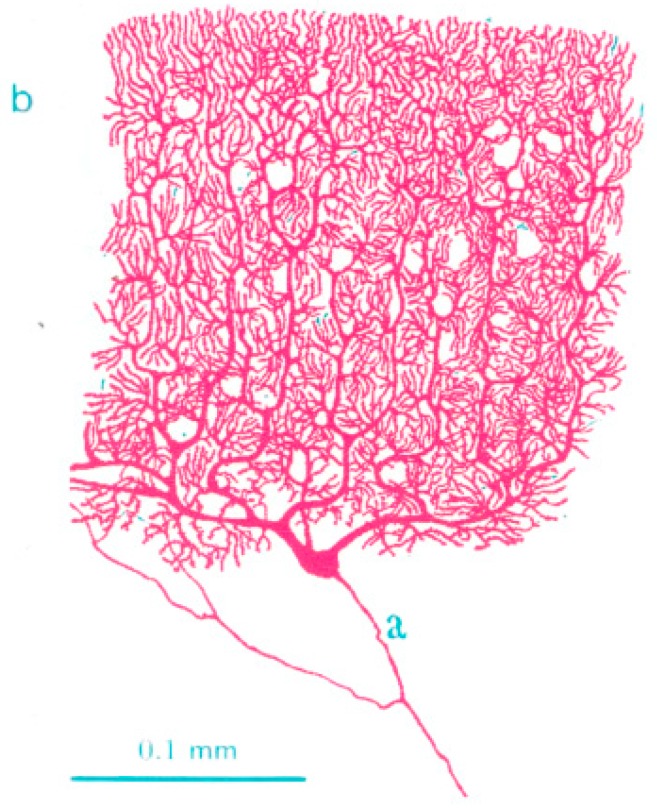
Inputs and outputs of a biological neuron where inputs (dendrites) are denoted as “b” in the figure and outputs (axons) are denoted as “a”. Since one synapse is connected at each input (dendrite), a huge number of synaptic weights are composed at a neuron.

**Figure 2:**
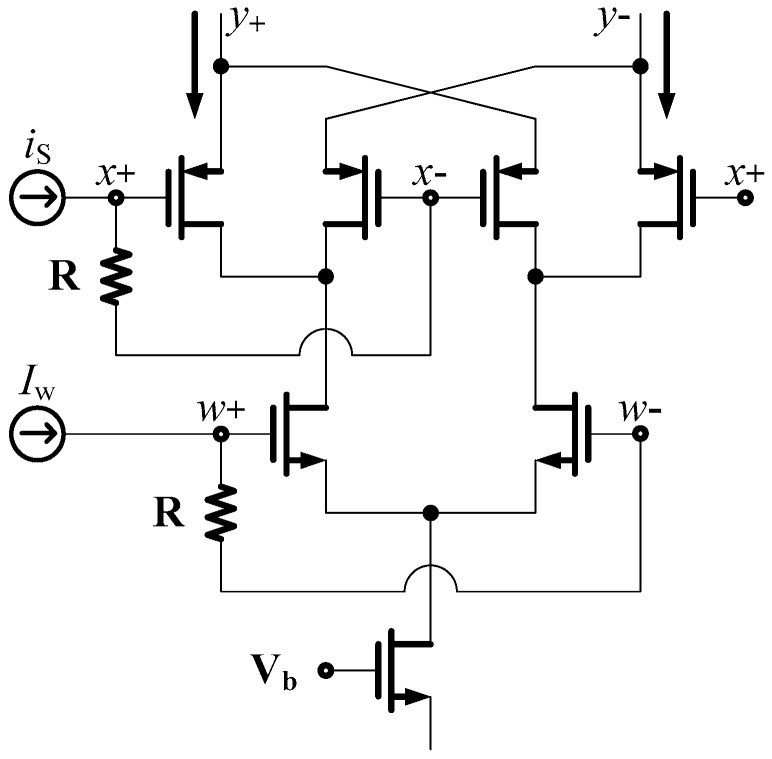
An analog multiplier employed to implement Cellular Neural Networks [17,18].

In biological neural systems, each neuron is connected to other neuron through a synapse between them. A synapse is a very special place in a neural cell, where memory and analog multiplication are performed. Figure 1 shows input and output connections of a biological neuron where inputs (dendrites) are denoted as “b” and output (axon) is denoted as “a”. Typically, about 10,000 inputs (dendrites) are connected at a single neuron. Thus, the same number of synaptic weights is needed in a neuron. Therefore, the implementation of huge number of neural synapses in a chip is a big roadblock for the development of an artificial neuron system. To make matters worse, one synaptic circuit is composed of many transistors as shown in Figure 2. Therefore, the implementation of an artificial neural system that mimics a biological neural system is seemingly far beyond our reach with the current circuit technologies.

**Figure 3:**
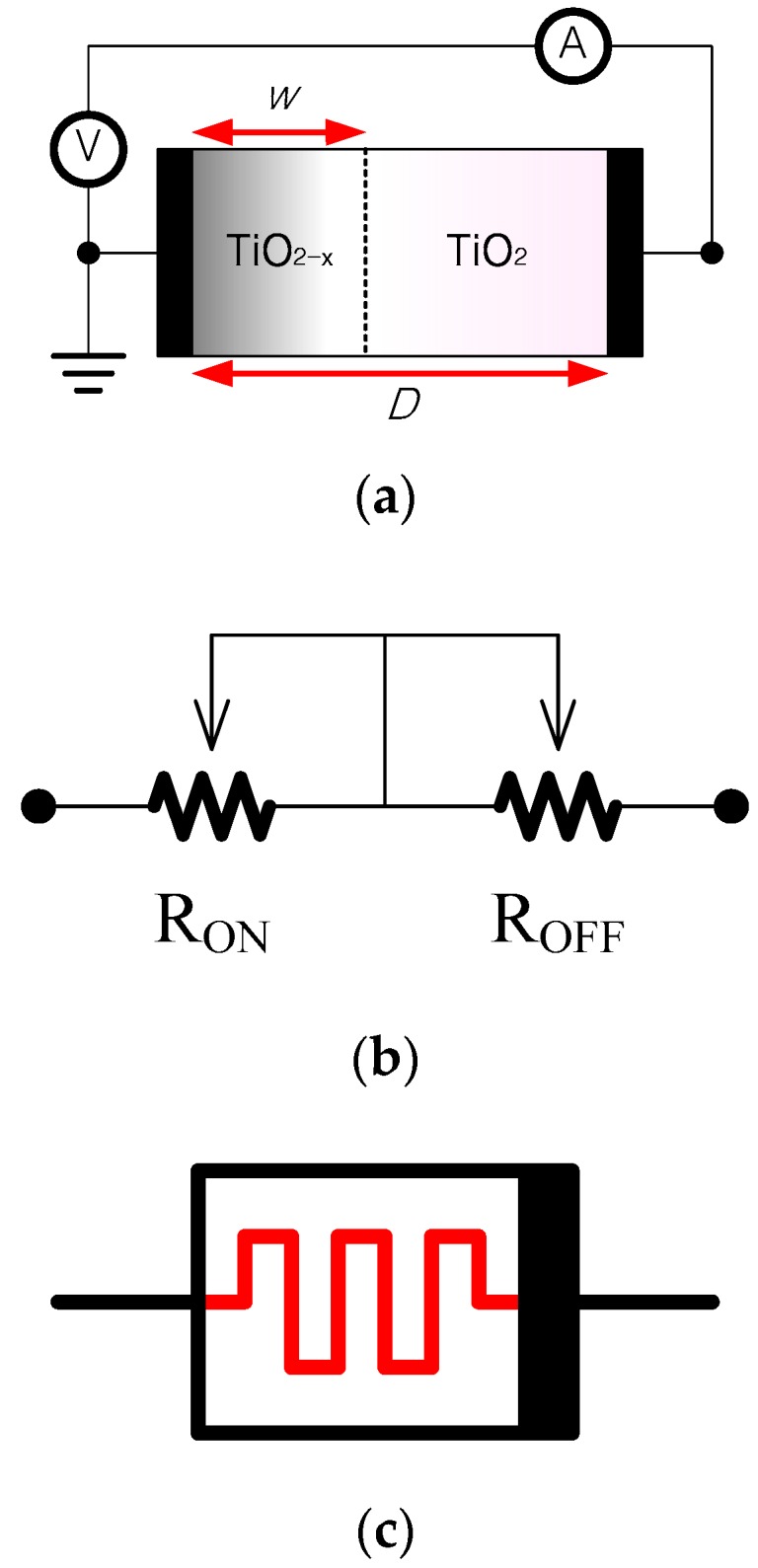
(**a**) Structure of TiO_2_ memristor, TiO_2−x_ and TiO_2_ layers are sandwiched between two platinum electrodes. When a voltage/current is applied, its memristance is altered; (**b**) equivalent circuit and (**c**) symbol of the Memristor.

Figure 3 shows a structure of a memristor fabricated successfully by HP [2]. In an HP TiO_2_ (Titanium dioxide) memristor model [2], an undoped region with highly resistive TiO_2_ and a doped region with a highly conductive oxygen vacancy TiO_2−x_ layer are sandwiched between two platinum electrodes. When a voltage or current signal is applied to the device, the border line between the doped and undoped layers shifts as a function of the applied voltage or current. In consequence, the resistance between the two electrodes is altered. Figure 3b,c are equivalent circuits.

In the TiO_2_ memristor, a thin titanium dioxide (TiO_2_) layer and a thin oxygen-poor titanium dioxide (TiO_2−x_) layer are sandwiched between two platinum electrodes. When a voltage or current is applied to the device, the resistance between the two electrodes is altered.

The memristor is defined [7] by

(1) $$v(t)=R(t)i(t)=\frac{d\varphi }{dt}\cdot \frac{dt}{dq}i(t)$$

where *φ*(*t*) and *q*(*t*) denote the flux and charge, respectively, at time *t*. Thus, the resistance can be interpreted as the slope at the operating point $q=q_{Q}$ at time *t* on the memristor $\varphi_{\_}q$ curve. If the $\varphi_{\_}q$ curve is nonlinear; the resistance will vary with the operating point.

Since the flux *φ* is defined by $\varphi \left(t\right)=\int_{-\infty }^{t}v\left(\tau \right)d\tau$, the resistance of the memristor, called the memristance, M, can be controlled by applying a voltage or current signal across the memristor, where

(2) $$R=M={\frac{d\varphi }{dq}|}_{\left(q_{Q}\cdot \varphi_{Q}\right)}$$

Equation (2) shows that a memristor is a kind of resistor that is variable depending upon an operation point on a charge and flux plane. In this sense, it is a programmable resistance.

**Figure 4:**
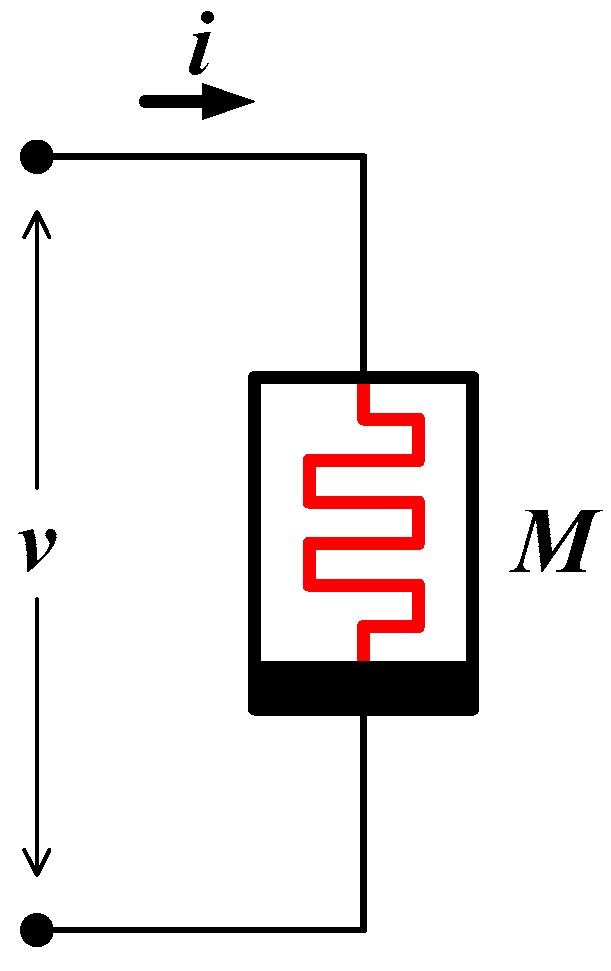
Memristor as an ideal element for a neural synapse where voltage across the memristor is a multiplication between an input current and a memristance.

Let the input of a memristor be a current and the voltage across the memristor be the output of a single memristor circuit as shown in Figure 4. Then, according to Ohm’s law, the voltage output is an analog multiplication between the current input and the resistance of a memristor as in Equation (3).

(3) v=i×M

Therefore, memristor is, in fact, an analog multiplier. Note that the resistance of a memristor is distinguished from that of an ordinary resistor in the sense that its resistance is programmable. It follows that the resistance of a memristor is called memristance. Since the memristance of a memristor can be altered (programmed) by input voltage/current and an analog multiplication is performed within a single device, the memristor is known as an ideal element for the implementation of synapses.

## 3. Nonlinearity in Memristor

In the TiO_2_ memristor, a thin TiO_2_ layer and a thin oxygen-poor TiO_2−x_ layer are sandwiched between two platinum electrodes. The TiO_2_ layer and the TiO_2−x_ layer are referred to as un-doped, and doped layers, respectively. When a voltage or current is applied to the device, the dividing line between the TiO_2_ and TiO_2−x_ layers shifts as a function of the applied voltage or current. As a result, the resistance between the two electrodes is altered.

Let *D* and w denote the thickness of the sandwiched area and the doped area (oxygen deficient area) in the TiO_2_ memristor, respectively, and let $R_{ON}$ and $R_{OFF}$ denote the resistances at high and low dopant concentration areas, respectively.

The relationship between the flux and the charge of the TiO_2_ memristor is given by [2]

(4) $$\varphi (t)=R_{off}\left\{q(t)\left[1+\frac{w_{0}}{D}\left(\frac{R_{ON}}{R_{OFF}}-1\right)\right]-\frac{\mu_{v}R_{ON}}{2D^{2}}\left(1-\frac{R_{ON}}{R_{OFF}}\right)q{\left(t\right)}^{2}\right\}+\varphi_{0}$$

where *µ_ν_* is the dopant mobility and w(t)/D is the state variable *x*.

The memristance M of a memristor can be computed with $M=\frac{d\varphi }{dq}$ as

(5) $$M=\frac{d\varphi }{dq}=R_{off}\left\{\left[1+\frac{w_{0}}{D}\left(\frac{R_{ON}}{R_{OFF}}-1\right)\right]-\frac{\mu_{v}R_{ON}}{D^{2}}\left(1-\frac{R_{ON}}{R_{OFF}}\right)q\left(t\right)\right\}$$

Assume that the applied input is a current *i*(*t*), then, Equation (5) is

(6) $$M=R_{off}\left\{\left[1+\frac{w_{0}}{D}\left(\frac{R_{ON}}{R_{OFF}}-1\right)\right]-\frac{\mu_{v}R_{ON}}{D^{2}}\left(1-\frac{R_{ON}}{R_{OFF}}\right)\int i(t)dt\right\}$$

The derivative of Equation (6) with respect to time gives

(7) $$\frac{dM(t)}{dt}=-R_{off}\frac{\mu_{v}R_{ON}}{D^{2}}\left(1-\frac{R_{ON}}{R_{OFF}}\right)i(t)$$

It follows from Equation (7)

(8) $$M\left(t\right)\frac{dM\left(t\right)}{dt}=-R_{off}\frac{\mu_{v}R_{ON}}{D^{2}}\left(1-\frac{R_{ON}}{R_{OFF}}\right)v(t)$$

Assume that a constant voltage *V* is applied as an input voltage. Integrating both sides of Equation (8) results in

(9) $$\frac{M{\left(t\right)}^{2}}{2}=C-R_{off}\frac{\mu_{v}R_{ON}}{D^{2}}\left(1-\frac{R_{ON}}{R_{OFF}}\right)V\cdot t$$

where *C* is the constant of integration.

It follows from Equation (9) that M(t) can be written as,

(10) $$M\left(t\right)=\sqrt{2\left\{C-R_{off}\frac{\mu_{v}R_{ON}}{D^{2}}\left(1-\frac{R_{ON}}{R_{OFF}}\right)V\cdot t\right\}}$$

Equation (10) exhibits a fact that memristance is a nonlinear function of time *t*. Thus, programming the memristor with an arbitrary value is very difficult.

Differently from the linear drift model described above, the nonlinear phenomenon appears often at the boundaries of nano-scale devices; with even the small voltage applied across nanometer devices, a large electric field is produced, and, therefore, the ion boundary position is moved in a non-linear fashion in nano-scale devices [14].

Several different types of nonlinear memristor models have been investigated [11,19,20]. One of them is the window model in which the state equation is multiplied by window function $F_{p}\left(w\right)$, namely

(11) $$\frac{dw(t)}{dt}=\mu_{v}\frac{R_{ON}}{D}i(t)F_{p}(w)$$

where *p* is an integer parameter and $F_{p}\left(w\right)$ is defined by

(12) $$F_{p}(w)=1-{\left(2\frac{w}{D}-1\right)}^{2p}$$

This is called the nonlinear drift model or memristive model. It is difficult to find the solution satisfying both Equations (11) and (12) analytically. However, w(t) can be computed numerically as

(13) $$w(t+\Delta t)=\mu_{v}\frac{R_{ON}}{D}\left(1-{\left(2\frac{w}{D}-1\right)}^{2p}\right)\Delta q+w(t)$$

where Δ*q* is the charge increment fed to the memristor during the time interval Δ*t* and computed by integrating the input current as

(14) Δq=∫I(t)dt=IΔt

Substituting the value of w from Equation (7) in Equation (8), we get

(15) $$M\approx \frac{R_{ON}}{D}w_{0}\left(1-\frac{R_{OFF}}{R_{ON}}\right)+\frac{R_{ON}}{D}K\Delta q\times F_{p}(w)\left(1-\frac{R_{OFF}}{R_{OFF}}\right)$$

The current voltage relationship can be obtained as

(16) $$v(t)=\left\{\frac{R_{ON}}{D}w_{0}\left(1-\frac{R_{OFF}}{R_{ON}}\right)+\frac{R_{ON}}{D}K\Delta q\times F_{p}(w)\left(1-\frac{R_{OFF}}{R_{OFF}}\right)\right\}i(t)$$

**Figure 5:**
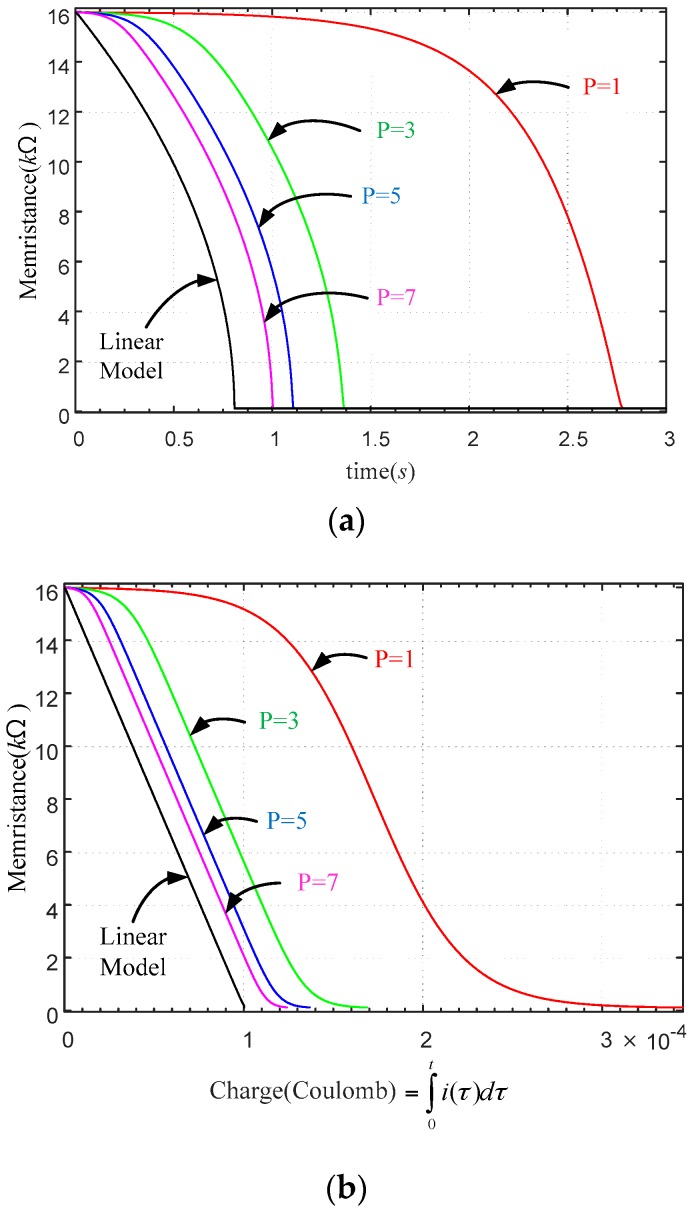
Memristance variation of a nonlinear memristor of (Equation of Window function) about: (**a**) time; (**b**) charge when a rectangular pulse is applied.

Figure 5 shows the graphs of the memristance vs. time and the memristance vs. time charge of the nonlinear models of memristors when a rectangular pulse is applied. The memristance is more nonlinear about time than that of charge. In addition, as the number *p* becomes smaller, the nonlinearity increases. On the other hand, as the integer p increases, the model tends to the linear model.

## 4. Linearization in Memristor Programming with Anti-Serial Architecture

**Figure 6:**
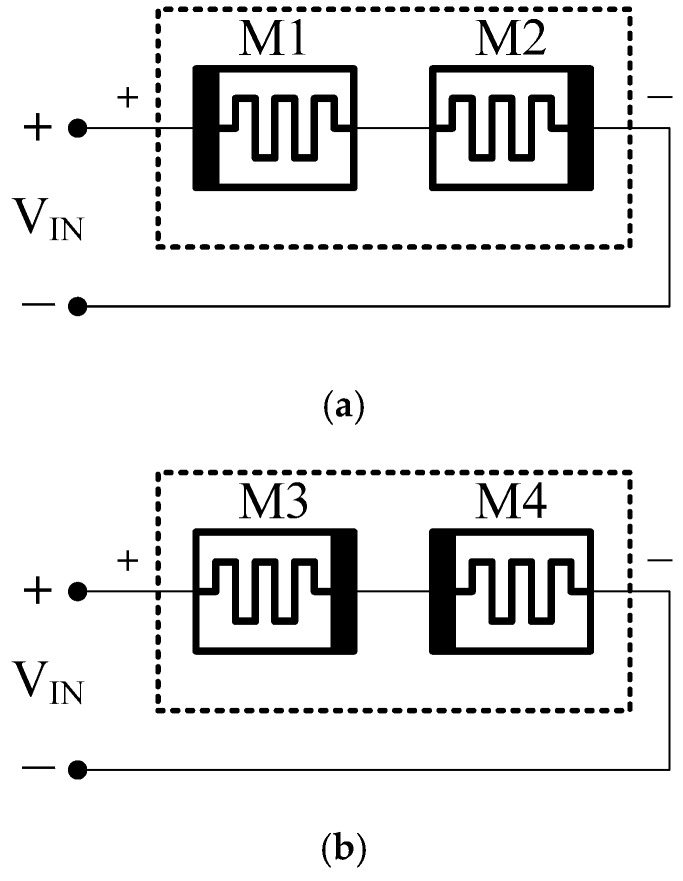
Memristor circuit with two memristors connected anti-serially. (**a**) M1 and M2 connected back to back in series; (**b**) serial connection with opposite polarities using M3 and M4.

Anti-serial memristor circuit is a circuit of two memristors in serial connection with opposite polarities as shown in Figure 6. When a positive voltage (or current) signal is applied to a circuit with two memristors connected in series, but with opposite polarities, then the memristance of M1 decreases, whereas the memristance of M2 increases. As a result, the composite memristance becomes constant, due to their complementary action [21].

Let us assume that the polarity of M1 is the same as that of the predefined reference polarity, and the polarity of M2 is opposite to that of the reference. If charge *q*(*t*) is injected into the positive terminal of the composite device, it acts as positive charge for M1, whereas it acts as negative charge for M2. Thus,

(17) $$q_{2}(t)=-q(t)$$

Similarly, the sign of flux $\varphi_{2}(t)$ is opposite to the reference, i.e.,

(18) $$\varphi_{c2}(t)=-\varphi_{2}(t)$$

Thus, flux $\varphi_{1}(t)$ and $\varphi_{2}(t)$ of memristor M1 and M2 can be written as functions of charge *q*(*t*), as

(19) $$\begin{array}{cc} \varphi_{1}(t)=R_{off} & \{q(t)\left[1+\frac{w_{01}}{D}\left(\frac{R_{on}}{R_{off}}-1\right)\right]\\ & -\frac{\mu_{v}R_{on}}{2D^{2}}\left(1-\frac{R_{on}}{R_{off}}\right)q{\left(t\right)}^{2}\}+\varphi_{1}(0) \end{array}$$

(20) $$\begin{array}{cc} \varphi_{2}(t)=R_{off} & \{q(t)\left[1+\frac{w_{02}}{D}\left(\frac{R_{on}}{R_{off}}-1\right)\right]\\ & +\frac{\mu_{v}R_{on}}{2D^{2}}\left(1-\frac{R_{on}}{R_{off}}\right)q{\left(t\right)}^{2}\}-\varphi_{2}(0) \end{array}$$

The total flux $\varphi_{C}(t)$ is the sum of $\varphi_{1}(t)$ and $\varphi_{2}(t)$.

When two memristors are assumed to be identical, and they are in the stable composite memristance state, the flux of the composite memristor becomes

(21) $$\varphi_{C}(t)=2R_{off}q(t)\left[1+\frac{w_{0}}{D}\left(\frac{R_{on}}{R_{off}}-1\right)\right]$$

where $w_{01}=w_{02}=w_{0}$. Furthermore, the memristance of the composite memristor can be obtained by differentiating Equation (21) with respect to *q*(*t*), as

(22) $$M_{C}=\frac{d\varphi_{C}(t)}{dq(t)}=2\left\{R_{off}\left[1+\frac{w_{0}}{D}\left(\frac{R_{on}}{R_{off}}-1\right)\right]\right\}=2M_{O}$$

where, $M_{C}$ is the composite memristance, and $M_{0}$ is a constant value of $R_{off}\left[1+\frac{w_{0}}{D}\left(\frac{R_{on}}{R_{off}}-1\right)\right]$.

Note that $M_{C}$ in Equation (22) is a constant, since all the related parameters of $M_{0}$ are constant.

Let M2 is a target memristor to program. From Equation (6), the expression of memristance *M_Target_* is

(23) $$M_{\text{target}}=R_{off}\left\{\left[1+\frac{w_{0}}{D}\left(\frac{R_{ON}}{R_{OFF}}-1\right)\right]-\frac{\mu_{v}R_{ON}}{D^{2}}\left(1-\frac{R_{ON}}{R_{OFF}}\right)\int i(t)dt\right\}$$

when a rectangular voltage pulse with V volt is applied, current *i*(*t*) during a non-zero pulse period can be computed as

(24) $$i\left(t\right)=\frac{V}{M_{O}}$$

Plugging Equation (24) into Equation (23), we obtain

(25) $$M_{\text{target}}=R_{off}\left\{\left[1+\frac{w_{0}}{D}\left(\frac{R_{ON}}{R_{OFF}}-1\right)\right]-\frac{\mu_{v}R_{ON}}{D^{2}}\left(1-\frac{R_{ON}}{R_{OFF}}\right)\frac{V}{M_{O}}t\right\}$$

All the parameters of the right side of Equation (25) are constant except time *t*. Therefore, it is a linear function of time. Comparing Equations (25) with (10) which is a nonlinear equation about time *t*, programing a memristor with Equation (25) is much easier than with Equation (10).

**Figure 7:**
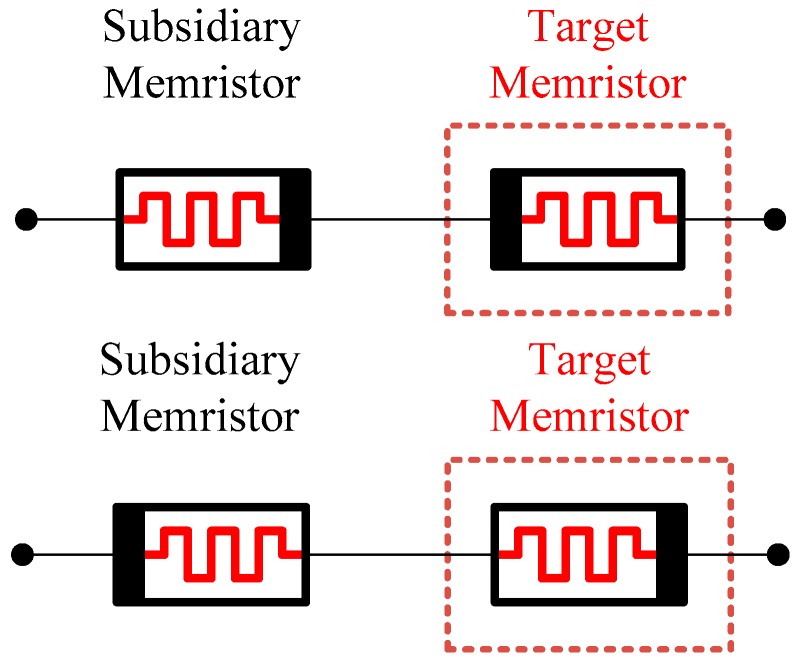
Proposed linearized programming method with anti-serial architecture. It is a circuit of two memristors in serial connection with opposite polarities. Though the individual behavior of memristance variation of two memristors is nonlinear about time, it becomes linear in an anti-serial connection due to the complementary action of two memristors with opposite polarities.

To program a target memristor with this method, the same type of a subsidiary memristor is prepared and connected to the target memristor in series with opposite polarity as in Figure 7. Since composite memristance of the anti-serial circuit is a constant value, the current through the circuit is constant. It follows that the memristance variation of the individual memristor is a linear function of pulse width since the memristance variation is a linear function of charge.

## 5. Application of the Anti-Serial Memristor Architecture to the Weight Programming of a Memristor Bridge Synapse

Anti-serial connections of memristor circuit are utilized to build a memristor bridge weighting circuit [7], which can be programmed linearly due to the cooperation of two sets of anti-serial memristor circuits.

**Figure 8:**
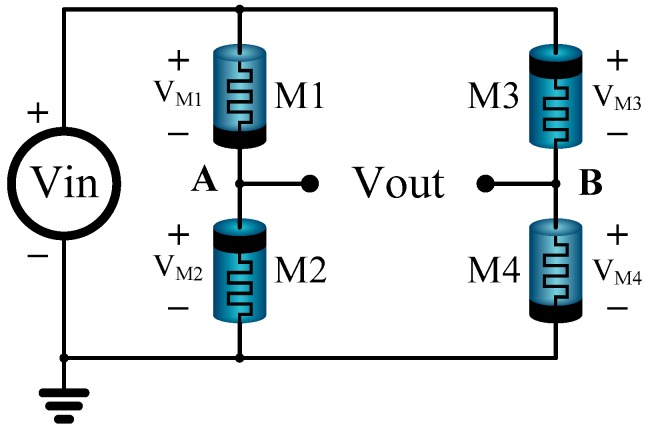
An application of the proposed anti-serial memristor circuit to the implementation of neural networks. With two different types of anti-serial memristor circuits, synaptic weights of neural networks can be programmed linearly about time when a direct current DC voltage signal is applied for the programing of a neural synapse.

The memristor bridge circuit consists of four identical memristors with different polarities indicated in Figure 8. When a positive or a negative pulse $V_{in}\left(t\right)$ is applied at the input, the memristance of each memristor is increased or decreased linearly depending upon its polarity. For instance, when a positive pulse is applied as input, the memristances of M1 and M4 (whose polarities are forward-biased) will decrease. On the other hand, the memristances of M2 and M3 (whose polarities are reverse-biased) will increase. It follows that the voltage $V_{A}$ at *node A* (with respect to ground) becomes larger than the voltage $V_{B}$ at *node B* for a positive input signal pulse. In this case, the circuit produces a positive output voltage $V_{out}$ representing a positive synaptic weight.

On the other hand, when a negative strong pulse is applied, the memristances are varied in the opposite direction and the voltage at *node B* becomes larger than that at *node A*. In this case, the circuit produces a negative output voltage $V_{out}$ representing a negative synaptic weight.

## 6. Simulation

The linearity in programing with several memristor circuits such as single and anti-serial circuit has been tested. The memristor models employed for these simulations are a linear drift model of HP TiO_2_ [2] and a nonlinear model with window function with *p* = 1 [16].

When we want to program a memristor to a certain memristance value, one of the easiest ways is by applying a constant voltage or current for a certain length of time. If memristance variation about time is linear, the desired value of memristance can be programmed easily since the programmed memristance would be proportional to the width of a voltage pulse.

**Figure 9:**
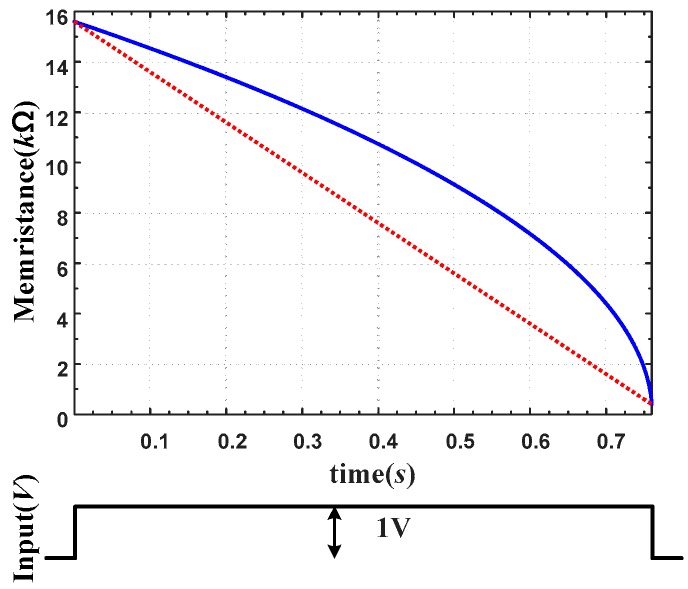
Nonlinear variation of the memristance about time of TiO_2_ memristor model when a constant voltage (rectangular pulse) is applied.

Figure 9 shows the memristance variation about time when a constant voltage of 1 V is applied to a TiO_2_ memristor model. Though the memristance curve is linear about charge, it is non-linear about applied time (pulse width) as shown with a solid line in Figure 9. Such a nonlinearity about time makes the programing of a memristor difficult. The desirable memristance curve is the dotted line.

**Figure 10:**
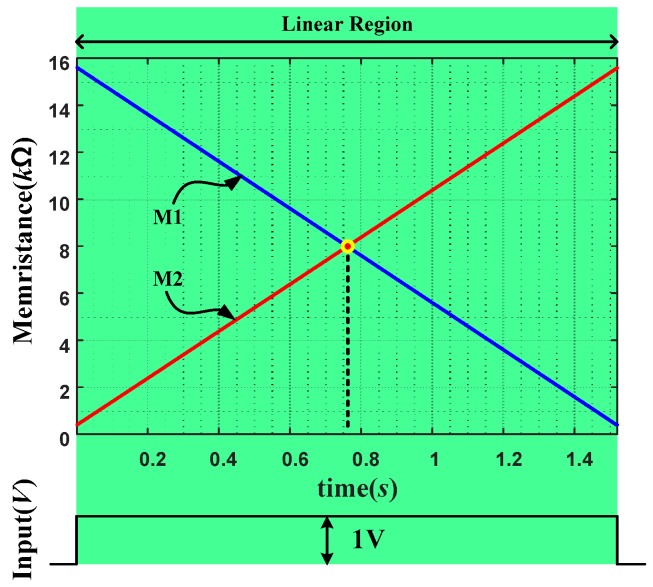
Linear variation of memristance when a constant voltage source is applied to the proposed anti-serial connection of two memristors.

Figure 10 shows a memristance variations of two memristors in the proposed anti-serial connection in Figure 7 when a constant voltage source is applied. Upon applying a constant voltage source to the anti-serial circuit, the subsidiary and target memristors are cooperating in complimentary fashion. The resultant memristance variation is linearized about time as shown in the figure.

Simulations to demonstrate the linearity in programming the memristor bridge synapse circuit have also been performed. If the memristance variation of each memristor of anti-serial memristor circuit is linear, the voltage change at each node of the circuit is supposed to be linear. In consequence, the weight of the memristor bridge synapse can be programmed linearly since the memristor bridge synapse circuit is composed of two different sets of anti-serial circuits.

**Figure 11:**
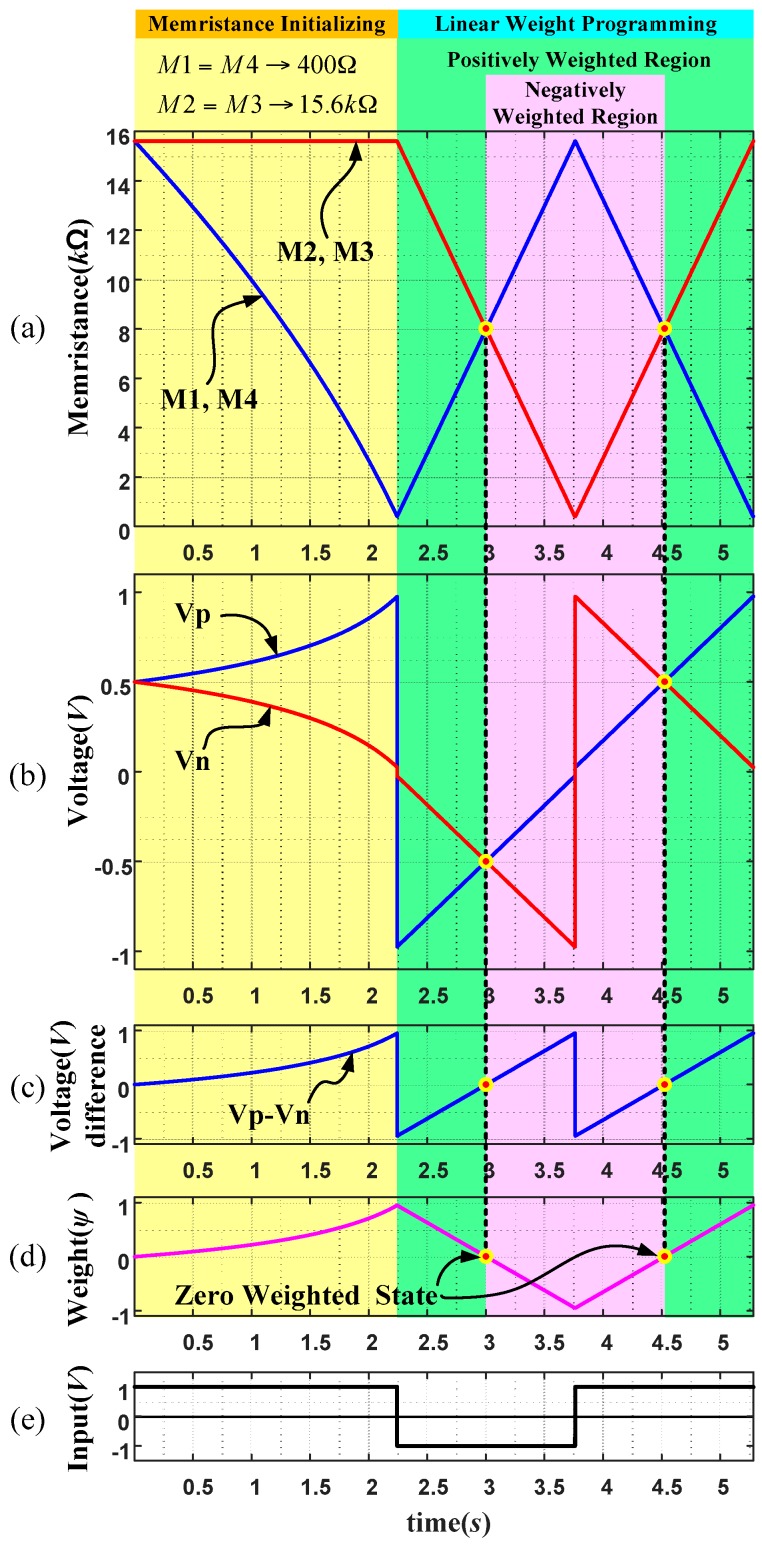
Linear programming of the memristor bridge synapse that is composed of two different types of anti- serial linear memristors. (**a**) variation of memristances; (**b**) changes of voltages *Vp* and *Vn*; (**c**) difference of middle voltage (*Vp* − *Vn*); (**d**) weight changes of the memristor bridge synapses; and, (**e**) wide pulse for programming.

Figure 11 shows a weight programming scenario where Figure 11a is the changes of two voltages (*Vp* and *Vn*) at middle points starting with 15.6 kΩ. When +1 V is applied for a long time as in Figure 11e, memristances of M1 and M4 are reduced gradually until 400 Ω is reached while M2 and M3 are kept with its highest memristance 15.6 kΩ. The change of memristance is a nonlinear function about time during this period. Then, when −1 V is applied afterwards, the memristances of M2 and M3 are reduced linearly while those of M1 and M4 are increased until M2, M3 and M1, M4 reach the minimum and maximum values, respectively. Then, when +1 V is applied again, the memristances of M2 and M3 are increased while those of M1 and M4 are decreased linearly.

Figure 11b shows the changes of voltages *Vp* and *Vn* which are the voltages at middle points of two anti-serial circuits during the period of Figure 11a. As seen in the middle of the figure, voltage changes linearly about time due to the linear change of memristances in Figure 11a. As the result, the voltage difference of middle voltage (*Vp* − *Vn*) is also linear as in Figure 11c and finally, weight changes of the memristor bridge synapses becomes also linear as in Figure 11d.

**Figure 12:**
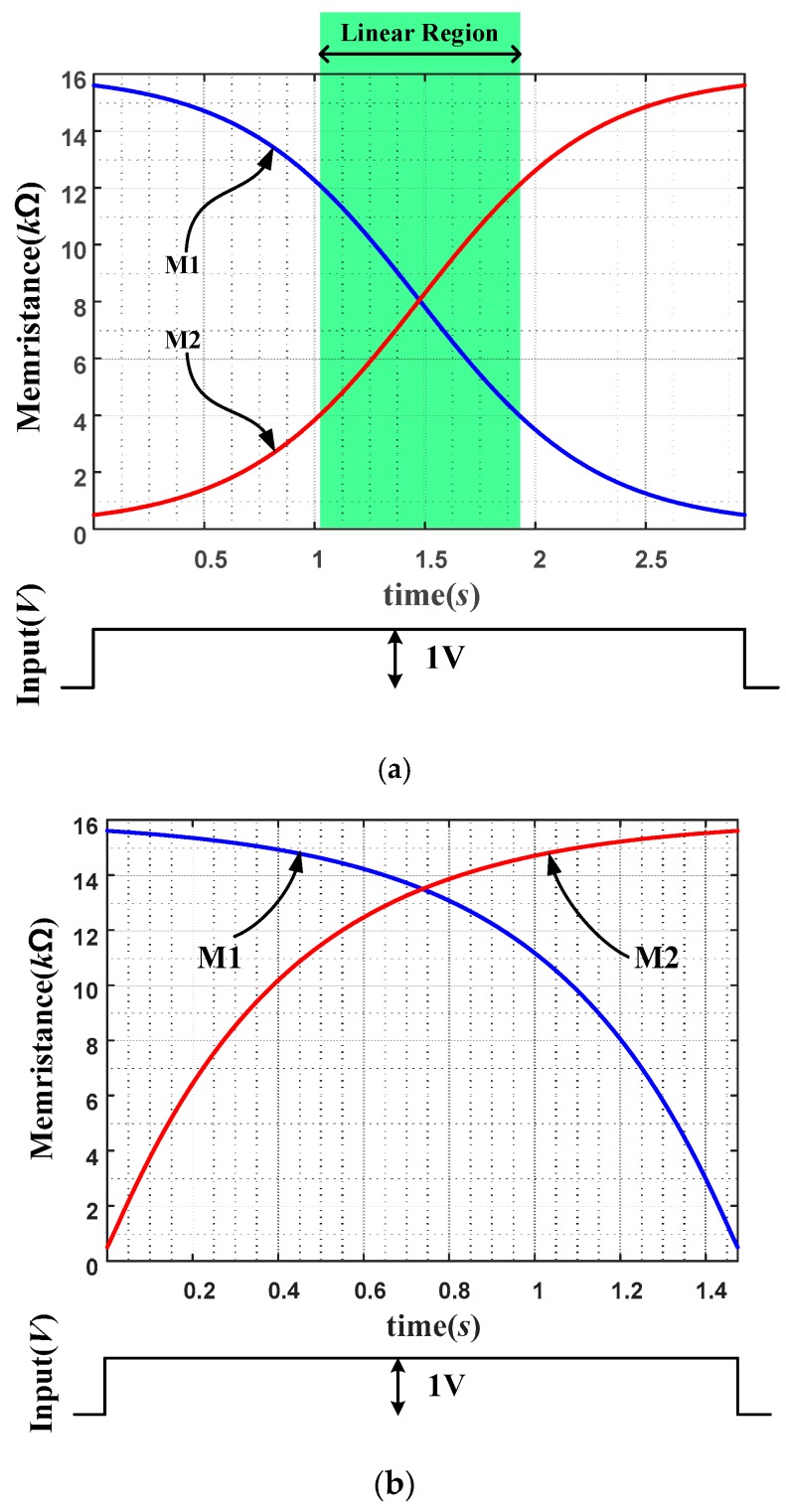
Linearized variation of memristance at the middle of the graph when a constant voltage source is applied at an anti-serial connection of two nonlinear memristors (**a**) and nonlinear variation of memristance of individual memristors for comparison when the programming signal is applied at each memristor individually (**b**).

The effect of the nonlinearity of memristor models to the performance of our memristor circuit has also been investigated. Figure 12a shows the memristance variations for each memristor of anti-serially connected non-linear memristor circuit. It is assumed that M1 and M2 have initial values of 15.6 kΩ and 400 Ω, respectively. When a positive DC input with +1 V is applied at the anti-serial circuit, the memristance of M1 is reduced and that of M2 is increased. Observe the region at the middle where graphs are almost linear though some nonlinear regions can be seen at the end of the curves. Since weighting operations are performed only in the linear region at the center as indicated with a box, linear programming can be performed with the proposed anti-serial architecture. Meanwhile Figure 12b is the variations of memristances when the programming signal is applied at individual memristors. As seen in the figure, the memristance changes as functions of time are highly nonlinear in all the range of the curves.

Comparing Figure 12a and Figure 12b, the memristances of anti-serial memristors are linearized significantly than those without the anti-serial connection.

**Figure 13:**
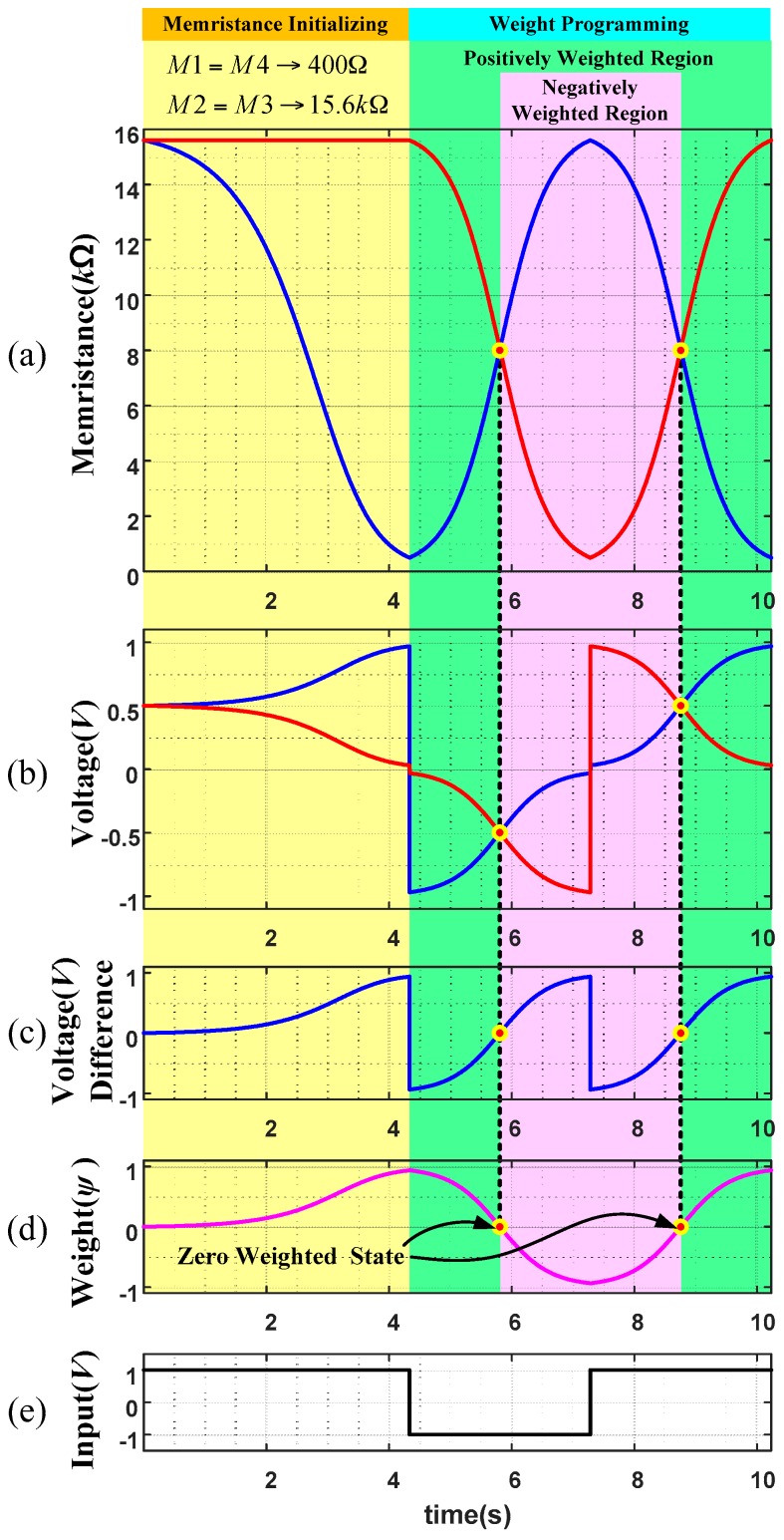
Linearized programming of the memristor bridge synapse which is composed of two different types of anti-serial non-linear memristors. (**a**) variation of memristances; (**b**) changes of voltages *Vp* and *Vn*; (**c**) difference of middle voltage (*Vp* − *Vn*); (**d**) weight changes of the memristor bridge synapses; and, (**e**) wide pulse for programming.

The effect of weight programming of memristor bridge synapses with non-linear model of memristors is also investigated. Figure 13 shows parameter variation of a memristor bridge synapse while the programming input signal as in Figure 13e is applied. All the necessary arrangements are the same as the linear memristor case in Figure 11 except that nonlinear memristors are employed.

Observe the memristance especially in Figure 13a and weight variation in Figure 13d at the time periods of [5.355, 6.247] s and [8.318, 9.209] s are all linearized very much.

## 7. Conclusions

In neuromorphic applications of memristors, a linear programming of memristance about time is important. In this paper, we proposed a method utilizing an anti-serial architecture.

Anti-serial architecture is a serial connection of two memristors with opposite polarities. It exhibits linearization in programming due to a complimentary action of two memristors; when the memristance of one memristor increases, the other decreases. Since composite memristance of the anti-serial circuit is a constant value, the current through the circuit is constant. It follows that the memristance variation of the individual memristor is a linear function about pulse width since the memristance variation is a linear function of charge.

Our proposed idea of linear programming of a memristor is by employing an additional subsidiary memristor with an opposite polarity when programming a target memristor is needed. When programming a target memristor is needed, a subsidiary memristor with an opposite polarity is prepared so that the subsidiary and target memristors construct an anti-serial architecture.

The validity of the proposed idea has been proved with linear drift model of HP TiO_2_ memristor. In addition, it has been applied in building a memristor synapse circuit that is composed of two different sets of anti-serial architectures. Due to the anti-serial architecture, weights have been programmed linearly about applied pulse width.

The proposed architecture has also been tested with memristor models of highly nonlinear characteristics. Memristances of the anti-serial memristor circuits and weights of memristor bridge synapse circuits are all linearized significantly around zero memristance and zero weight regions, respectively.
